# Priority setting in the provincial health services authority: survey of key decision makers

**DOI:** 10.1186/1472-6963-7-84

**Published:** 2007-06-12

**Authors:** Flora Teng, Craig Mitton, Jennifer MacKenzie

**Affiliations:** 1 Centre for Healthcare Innovation and Improvement, B.C. Research Institute for Children's and Women's Health, Vancouver, Canada; 2 Faculty of Health and Social Development, University of British Columbia Okanagan, Kelowna, Canada; 3 Provincial Health Services Authority of B.C., Vancouver, Canada

## Abstract

**Background:**

In recent years, decision makers in Canada and elsewhere have expressed a desire for more explicit, evidence-based approaches to priority setting. To achieve this aim within health care organizations, knowledge of both the organizational context and stakeholder attitudes towards priority setting are required. The current work adds to a limited yet growing body of international literature describing priority setting practices in health organizations.

**Methods:**

A qualitative study was conducted using in-depth, face-to-face interviews with 25 key decision makers of the Provincial Health Services Authority (PHSA) of British Columbia. Major themes and sub-themes were identified through content analysis.

**Results:**

Priorities were described by decision makers as being set in an ad hoc manner, with resources generally allocated along historical lines. Participants identified the Strategic Plan and a strong research base as strengths of the organization. The main areas for improvement were a desire to have a more transparent process for priority setting, a need to develop a culture which supports explicit priority setting, and a focus on fairness in decision making. Barriers to an explicit allocation process included the challenge of providing specialized services for disparate patient groups, and a lack of formal training in priority setting amongst decision makers.

**Conclusion:**

This study identified factors important to understanding organizational context and informed next steps for explicit priority setting for a provincial health authority. While the PHSA is unique in its organizational structure in Canada, lessons about priority setting should be transferable to other contexts.

## Background

Due to limited resources, health care decision makers must make choices about what services to fund and what not to fund. This process of priority setting has traditionally been shaped by organizational cultures where norms and incentives have implicitly supported historically-based resource allocation processes [1]. That is, in most health care organizations, the process underlying decision making is based on the previous year's expenditure being rolled over to the current year, with some political and/or demographic adjustments. This can lead to 'allocation by stealth' and enables politics to directly enter into the fray [2]. The problem is, over the last decade, decision makers in various organizations across countries have expressed dissatisfaction with these processes, desiring more explicit, evidence based approaches to priority setting [3-6].

In order to move away from historical and/or politically driven allocation models, towards a more explicit, evidence-based process, knowledge of the organizational context is required [7,8]. The reason for this is two-fold. First, sustainability of a novel process is reliant on that process fitting with existing practices and beliefs. Second, understanding the context provides insight into whether a move towards an explicit priority setting process is appropriate or desired by a given set of decision makers. Knowledge about current decision making practices within health care organizations is thus pivotal to improving priority setting processes [2,7].

To better understand organizational context with respect to priority setting and to investigate the possibility of moving towards a more explicit process, a survey of key decision makers was conducted in the Provincial Health Services Authority (PHSA) of British Columbia, Canada. The objectives of this survey were: 1) to obtain insight into past organizational practices with respect to priority setting; 2) to identify strengths and weaknesses of past priority setting activity; 3) to determine strategies for improvement in priority setting practices; and finally 4) to determine likely barriers and facilitators in, and ultimate feasibility for, moving towards an explicit process for priority setting.

The purpose of this paper is to present key findings of this decision maker survey. The findings serve to expand our understanding of the organizational context within the PHSA, and through this, should provide insight into how other organizations function with respect to priority setting and resource allocation processes. This work builds on previous surveys that have been conducted elsewhere in Canada and in Australia [5,6], and parallels ongoing research with health service commissioning bodies in the United Kingdom.

## Methods

### Context

We conducted a survey of key decision makers from the PHSA and two of its member agencies, the British Columbia Children's Hospital and Mental Health Services. The PHSA was created in December 2001 and became fully operational late in 2002. The PHSA is unique from the other five health authorities in British Columbia (B.C.) with its provincial, rather than regional, mandate [9]. All six health authorities are allocated resources directly from the Provincial Ministry of Health to administer and deliver the majority of publicly funded health care services in the Province (i.e., there is no direct delivery of services from the Ministry, but physicians are paid on a fee for service basis from the Ministry and are under no contractual obligation to the health authorities). Approximately 70% of health care funds in Canada are generated through provincial and federal taxation and constitute the 'publicly funded' system, from which the health authorities receive their funding; the remaining 30% of 'private' funding is raised through out of pocket expenditure and largely employer-based private health care insurance.

As a provider of specialized services, the PHSA coordinates the activities of 8 provincial agencies: B.C. Cancer Agency, B.C. Centre for Disease Control, B.C. Children's Hospital and Sunny Hill Health Centre for Children, B.C. Provincial Renal Agency, B.C. Transplant Society, B.C. Women's Hospital & Health Centre, and B.C. Mental Health Services (Forensic Psychiatric Services Commission and Riverview Hospital). In addition, the PHSA is responsible for Cardiac Services, and the provincial coordination of emergency and surgical services. Each agency administers and delivers health care in their respective areas, while the PHSA serves as a provincial umbrella organization and provides corporate services across the agencies.

With the creation of the PHSA, the authority of each individual agency was transferred from the Ministry of Health to the PHSA. A Performance Agreement was signed which outlined the responsibilities of both organizations. The PHSA was mandated to achieve costs savings and manage with zero budget increases over the first three years [10]. The annual operating budget of the PHSA in 2003 was approximately $1.2 billion [11]. With budget pressures and growing health care costs, the PHSA was interested in exploring other options for priority setting. As a new organization with a unique provincial mandate and an expressed interest in priority setting, the PHSA was able to serve as a useful setting in which to better understand organizational context with respect to priority setting and to investigate the possibility of moving towards a more explicit process.

### Study design and sample

**Figure 1:**
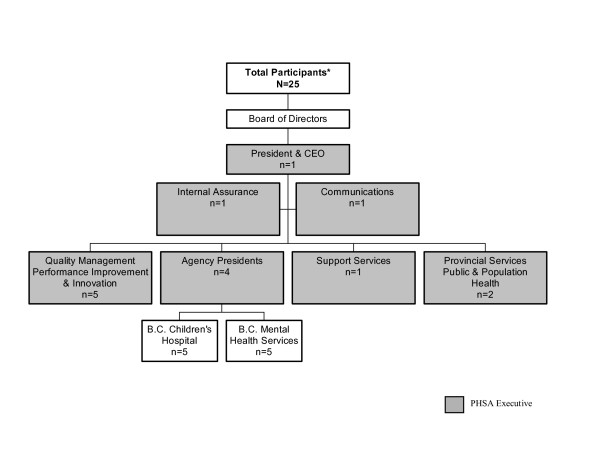
**Participants in the PHSA decision-maker survey**. * The PHSA Executive is comprised of members from the portfolios outlined in the gray boxes. The numbers shown here represent only those who participated in the study.

This is a qualitative study using data from in-depth, face-to-face interviews with key decision makers of the PHSA. All members of the PHSA Executive Team, plus the Internal Assurance Officer (n = 15), were invited and agreed to participate in the study (Figure 1). In addition, a further set of decision makers from two of the PHSA's eight agencies (B.C. Children's Hospital, n = 5; and Mental Health Services, n = 5) were also invited to participate on the basis of an expressed interest in the work from the respective Presidents of these two agencies and an expressed desire to examine their historical priority setting activity. For the Children's Hospital and Mental Health Services, a purposeful sampling strategy was employed [12], whereby a list of decision makers whose roles and responsibilities included priority setting was developed with input from a senior manager within the PHSA. Five members from each agency were invited to participate and all agreed. The 10 decision-makers from the agencies were asked their perspectives on the PHSA as a whole and on their views of decision-making within their own agencies, with only the data regarding the PHSA as a whole presented in this paper.

An initial letter describing the survey and requesting participation was sent to the potential participants. Written informed consent was obtained at the start of each interview. In total, the views of 17 administrators and 8 clinician administrators within the PHSA are presented in this study. Written notes were made during each interview and interviews were audiotaped with permission. The audiotapes were independently transcribed verbatim. The interviews were conducted between June and August 2004 by a research assistant, and thus reflect priority setting practice prior to this period. The Behavioural Research Ethics Board at the University of British Columbia (UBC) approved the study.

### Survey and analysis

The survey was adapted from previous surveys conducted in Australia and Alberta [1,5,13]. The interview guide comprised 15 questions (Table 1); the questions were asked in the order listed for each respondent. This survey represents the first step in a framework for describing, evaluating and refining priority setting activity as outlined by Martin and Singer [7]. The purpose of the survey was to understand the context within the PHSA as it related to priority setting activity, and to explore the potential for embarking on an explicit priority setting process within this organization.

**Table 1:** Interview guide

1	Can you describe for me the process that is currently used to identify priorities and allocate resources within the PHSA?
2	Overall, do you think the process works well? What are the strengths of the process?
3a	How well is the publicity condition met in this organization?*
3b	How well is the relevance condition met in this organization?*
3c	How well is the appeals condition met in this organization?*
3d	How well is the enforcement condition met in this organization?*
4	How can the current process of setting priorities and allocating resources be improved?
5	What types of information (or data or evidence) that are not currently used would you most want to use to improve decision making in setting priorities and allocating resources?
6	What barriers are currently faced in undertaking the priority setting process within the PHSA?
7	Noting the organizational culture of the PHSA, how would this environment respond to a move towards an explicit, more formal, process of priority setting?
8	How do the group dynamics at a typical executive meeting impact priority setting decisions?
9	What factors do you think are necessary for sustaining an explicit, more formal, priority setting process in the PHSA? Please be as specific as possible.
10	How has the public been used in priority setting/resource allocation processes in the past?
11	Ideally, how would you want the public to be involved in the priority setting process?
12	What role have physicians played in priority setting/resource allocation processes in the past?
13	Ideally, how would you want the physicians to be involved in the priority setting process?
14	How well do you think the values of the PHSA are incorporated into priority setting activity?
15	How should the values of the PHSA be incorporated into the priority setting process?

*These questions are based upon an ethical framework called Accountability for Reasonableness [19], with details of each ethical 'condition' presented to the respondents prior to eliciting their responses.

^1 ^Condition of relevance: Decisions should be made on the basis of reasons (i.e. evidence, principles, values, arguments) that 'fair-minded' stakeholders can agree are relevant under the circumstances; Publicity: Decisions and their rationales should be made available to stakeholders; Revision and appeals: There should be opportunities to revisit and revise decisions in light of further evidence or arguments, and there should be a mechanism for challenge and dispute resolution; Enforcement: There is a voluntary or regulatory mechanism for ensuring that the other three conditions are met. Condition of relevance: Decisions should be made on the basis of reasons (i.e. evidence, principles, values, arguments) that 'fair-minded' stakeholders can agree are relevant under the circumstances; Publicity: Decisions and their rationales should be made available to stakeholders; Revision and appeals: There should be opportunities to revisit and revise decisions in light of further evidence or arguments, and there should be a mechanism for challenge and dispute resolution; Enforcement: There is a voluntary or regulatory mechanism for ensuring that the other three conditions are met.

Feedback on the survey was obtained from the first decision maker interviewed. The researchers analyzed the interview transcripts using content analysis [14]. Major themes (e.g., 'stakeholder participation') and sub-themes (e.g., 'public involvement in priority setting) were developed through constant comparison and categorized [14]. Thus, each category was compared across the data set until no new categories were identified. Following the first interview, a list of codes was developed and the survey was refined for subsequent interviews.

During analysis, the code structure was refined once for consistency and clarity. Once categorized, the data were interpreted into meaningful concepts pertaining to current and desired priority setting practices in the PHSA. As categories of meaning emerged, the researchers searched for those that had internal convergence and external divergence [15]. That is, the categories were constructed so that they were internally consistent but distinct from one another. Using a consistent coding structure, a research assistant independently coded all transcripts. A second investigator coded a sample of the transcripts and the research team met to discuss the coding structure and analysis, with consensus reached in all cases were discrepancies arose.

## Results

The results are presented as follows: 1) current organizational practices; 2) strengths and weaknesses of priority setting activity to date; 3) strategies for improvement, particularly in relation to cultural change, stakeholder involvement, and fairness of process; and 4) barriers and facilitators in moving forward with an explicit approach to priority setting. The data presented reflects the opinions of key decision makers regarding priority setting at the macro-level of the PHSA. While data was also collected on the priority setting processes of the B.C. Children's Hospital and B.C. Mental Health services alone, these results are not presented here. The survey was designed to examine previous priority setting practices from the time the organization was constituted up to the time of the interviews (late 2002 – summer 2004).

### Current priority setting processes

The priority setting process occurs at the level of the Executive Committee in the PHSA. Decision makers within the Executive Committee indicated that the process of priority setting is largely based on 'the squeaky wheel getting the grease', and suggested that resources tend to go to 'whoever yells the loudest'. This is exemplified by the opinion of this decision-maker:

It's a squeaky wheel process. Whoever is able to more clearly articulate their problem, or lobby for their group or, through some other form of power and influence, impact whatever process is in place that year will come out with some outcome.

Priorities were described as being set in an ad hoc manner, with resources allocated to satisfy the most people and incur the least opposition. The decision makers noted that priority setting usually occurs in the context of the budget cycle and that the process is driven by historical allocation. One decision maker described the process as follows:

I don't think that I'm really aware of any mechanism to determine medium and long-term priorities for the PHSA. I think that it is possibly because of the newness of the organization and, in essence, the imperative for its creation, which clearly prioritized balanced budgeting and sustainability as being the key drivers of the short-term. So I think when it comes to things such as priority setting and allocation, it's really been determined more by managing activity to budget than it has been in terms of strategic outcomes in terms of health care.

Decision makers stated that there had been little discussion of resource re-allocation across PHSA agencies, with each agency by and large operating as its own entity. Decision making criteria had been used in the past to assess alternative investment proposals in some instances, but the criteria were not consistent throughout the PHSA. However, decision makers would routinely incorporate best practice information when assessing options. Overall, it was clear that decision-makers were dissatisfied with current priority setting processes and desire a better framework by which to make decisions. One decision-maker phrased it in this manner:

No, I don't think [the system] works well. I think it works as well as it can without some more overarching framework in which to make those decisions... When you get down to it, if the decisions are, 'Should we put more into cancer or should we put more into mental health?' – who at the end of the day should actually be making that decision? As medical people, one can bring forward the evidence for the benefit. In terms of the costing, etc., one can bring the cost-effectiveness. But who actually is the beneficiary to set the priority?

### Strengths and weaknesses

Decision makers identified a number of strengths in their priority setting practices. First, many respondents identified the creation of the Strategic Plan as a potential organizational strength. The year-long planning process incorporated both internal (i.e. employees) and external (i.e. other health authorities and the Ministry of Health) stakeholders and allowed them to come together to discuss the future directions of the PHSA. The aim of the strategic planning exercise was to establish a unified vision across the agencies. Decision makers viewed the plan as the first step towards a more "fair, open, and transparent" process. In theory, the goals outlined in the Strategic Plan were to created "to provide governance and direction to its agencies in order to achieve greater levels of efficiency and effectiveness through the consolidation of corporate services and to begin developmental work in coordinating province-wide services" [16]. The Strategic Plan was officially released in April 2004, only several months before this study was conducted.

Another strength identified by decision makers was the openness of the PHSA towards explicit priority setting. One decision maker expressed that, "despite the whining and the gnashing of teeth, I think we're ready to move to something that makes a little more sense". In addition, the strong research base of the organization is a strength that was noted, with a clear appreciation for evidence in both policy-making and clinical practice. One decision-maker stated, "I think the fact that we have such a strong basic and translational research infrastructure within many of our health care organizations within the PHSA is a real strength".

Several weaknesses were also identified through the interviews and are summarized in Table 2. Weaknesses categorized as 'systemic' refer to issues in the structure, policy, or systems of the organization, while those categorized as 'individual' refer to the attitudes and behaviour of individual decision makers. The categories of internal and external weaknesses refer to issues within and outside of the PHSA, respectively.

**Table 2:** Perceived weaknesses in priority setting in the PHSA

***Systemic***	**Internal**
	Central decision making creating a feeling of disempowerment among managers
	Lack of true accountability to conserve resources
	"Do it all" mentality that prevents the organization from identifying disinvestments
	Incentive to overspend because efficiency is not rewarded
	Lack of structural and cultural integration due to the recent creation of the PHSA
	**External**
	Confusion regarding role and authority of the BC Ministry of Health and the PHSA
	Limitations in priority setting due to provincial mandate and global priority setting
***Individual***	Lack of priority setting skills and tools which support resource re-allocation
	Unwillingness to release resources from own budgets to fund investments elsewhere
	Fear of being explicit in priority setting
	Decision makers jaded to change processes because of too much change in the institution
	Lack of management training for physician-leaders

One systemic, internal weakness was a lack of structural and cultural integration within the organization. This was attributed to the recent creation of the PHSA, and related to the challenge noted above of re-allocating resources across the agencies. In addition, decision makers said that there tended to be an organizational 'do-it-all' mentality, rather then an acceptance of needing to make overt rationing decisions. Another weakness noted by participants was a perceived lack of authority over program areas within the agencies. One decision maker preferred a structure where "individuals have a degree of autonomy and authority over their area of responsibility and have some flexibility within that area to move forward, rather than having to do everything at the most senior level".

A perceived weakness under the individual category was that decision makers would be unwilling to release resources from their own program budgets to fund investments elsewhere. As one decision maker described, "everybody thinks their business on this site is the most important, that it has to be done here. It's pretty hard to set priorities when everybody thinks their thing is the most important". Yet another weakness identified by many participants was a jaded attitude of decision makers towards new change processes. In response to the question of how decision makers would respond to explicit priority setting, one participant noted:

"I think people would be very jaded, to start with. It would need to be clear that people [are] just so fed up... So I think [an explicit process] would have to be very clear and would have to stand the test... It would have to show that there was open input and that people were able to make a difference."

### Strategies for improvement

Decisions makers identified several improvement strategies that would overcome the weaknesses in their priority setting process. The main area for improvement, noted by the participants, was a desire to have a process that was more transparent and defensible (Table 3). Decision makers suggested that such a process should take both context and politics into consideration. In addition, a vision for the process should be defined and clearly communicated to all stakeholders. Participants also suggested that goals, outcomes, and benchmarks for success should be defined, using the PHSA Strategic Plan as a guide. The consistent application of the process was also seen as integral to any plan. In addition, it was felt that any process should be time-sensitive and driven by evidence. As one decision-maker noted, there is a strong research base of the organization, but the use of this evidence could be improved.

**Table 3:** Strategies for improvement

*Increase transparency and accountability*	• Make the decision making process more transparent and accountable to internal and external stakeholders
*Create explicit process*	• Align process with organizational context and account for politics
	• Clearly communicate vision of the process to all stakeholders
	• Define goals, outcomes, and benchmarks for success incorporating the Strategic Plan
*Initiate cultural change*	• Create time-sensitive, evidence-driven process
	• Apply the process in a consistent manner
	• Provide education to create a culture of explicit priority setting
*Increase stakeholder involvement*	• Include public opinion at a general level and provide management training for physicians
*Enhance fairness*	• Create explicit appeals process for priority setting decisions

We have a strong base of research in all of our organizations [agencies], so there is probably more evidence out there about what works and what doesn't work than we currently use in our resource allocation practices."

Decision makers further stated a need for developing a culture that supports explicit priority setting. It was suggested that this could be achieved through education of internal stakeholders and the demonstration of real results. The former was viewed as a key component to increasing awareness about explicit priority setting, while the ability to demonstrate results was seen as a way to positively reinforce the benefit of a new process and contribute to its continued use.

While the PHSA currently uses stakeholder opinion in priority setting, decision makers believed that stakeholder involvement could be improved. Participants stated that the general public was not involved in priority setting to date. The main reason cited for this was the difficulty of finding the right forum to garner public opinion. One decision maker described this predicament:

The public can be important, unquestionably, because that's really the only way one can put social context around how taxpayers' dollars get spent. So I don't have any difficulty with that context. How you engage the public and what you ask the public becomes a very difficult issue for consideration, because one can't really hold a Town Hall Meeting and say, "Where would you like your money spent – on mental health or cancer?"

Decision makers believed that while it was important to obtain public opinion, the ideal role of the public would be involvement at a fairly general level. For example, it was felt that ascertaining the public's opinion on broad areas of importance would be more useful than input on specific decisions. Participants suggested that this could be done through surveys, public forums, focus groups, or having a member of the public at the decision making table. One decision maker described what they viewed as the ideal role for the public:

I think the public would have to be involved at a very high level in deciding what the general goals and values are that one makes a decision around. They need to say "this is what is important for them" and then leave it to decision makers to apply those values to their decision making process and its up to the board be the governors to ensure that decision makers are applying that on their behalf.

In addition to the role of the public, participants were also asked about the role of physicians in priority setting. Many decision makers believed that physician-stakeholders were quite involved in priority setting already, but that their involvement could be improved. The majority of decision makers felt that the ideal role of physicians would be to bring clinical evidence to the table. Participants also noted that physicians face an inherent conflict of interest. With a fee-for-service system, physicians have an incentive to utilize services rather than conserve resources. This incentive can create difficulties in allocating system resources in the most efficient manner. It was suggested that physician training in management practice would be useful.

Another area of improvement cited by decision makers was the issue of fairness in priority setting. In the PHSA, participants noted that most decisions were publicly announced, but the rationale and decision making process behind the decisions were not publicly available. Despite this, participants believed that as a whole decisions were data-driven. Many decision makers also noted that there was no formal mechanism for appealing allocation decisions. As a result, decision makers did not believe that adequate enforcement existed to ensure that decisions were made in a fair and equitable manner. Overall, decision makers believed that components of the priority setting process could be considered fair, but that further improvement was required.

### Barriers and facilitators for change

Despite the desire for greater transparency, decision makers identified a number of barriers that would hinder a move towards an explicit process based on the notion of re-allocating resources across service areas. One barrier was the mandate of providing specialized services. The PHSA is comprised of eight highly specialized agencies, which serve widely differing populations. With this mandate, decision makers must set priorities knowing that they are the only organization providing that service. Decision makers stated that setting priorities in this context can be quite difficult and that they do not feel they have the right tools to inform such decisions.

If you were to take something away from a place... it's not as though you could say, 'Okay, we're not going to do this at [Hospital A], but they can go to [Hospital B].' For the tertiary stuff that we do here, you can't do that, because there's no place else in the province. It's not as though we could say, 'Okay, we're not going to do that here, but somebody else will do it.' That, I think, is a significant barrier.

Other barriers identified in moving to an explicit priority setting process were a lack of shared vision in the PHSA, a lack of priority setting skills among the management team, and the lack of decision maker buy-in for such a move. In addition, decision makers noted that there was a lack of real or perceived authority to change the process and a significant political influence in priority setting. According to one decision-maker:

What tends to happen, I think, is that new programs get funded on the basis of politics, not on the basis of need or priority setting. So these last two or three years there's been money for autism – nothing to do with us; everything to do with politics. Five years before that it was eating disorders. Again, the politicians became involved and said, 'We must have an eating disorder program.' They didn't come to us and ask us what we wanted. They get very much involved in the micro-management and allocation process.

In addition, a lack of budget integration across agencies is a major barrier to explicit priority setting. On this issue, one decision maker stated that the PHSA has taken "a whole bunch of agencies and put them together, and all their budgets together with them." Participants commented that with the barriers of an historical structure, it is difficult to shift resources across agency lines.

To counterbalance the barriers for change, decision makers also highlighted several facilitators that would need to be fostered to aid in the implementation of an explicit priority setting process. These included a strong leadership team and commitment to explicit priority setting, as well as consistent application of the process, demonstrated results and an adequate amount of resources for re-allocation across services. One decision maker described the importance of strong leadership and commitment to priority setting:

I think fundamentally we have to have 100 percent commitment from the board and CEO. It's always the same. If they're not really committed to [it], then it's probably not going to be well endorsed.

In addition, a culture of openness to priority setting, a culture of learning, and a data-driven culture were cited as important facilitators that currently existed in the organization, which would assist in the implementation of an explicit priority setting process.

## Discussion

### Summary of findings

This study is the first to our knowledge to examine the views of health care decision makers on priority setting and organizational context in British Columbia. This study is also novel in its examination of a provincially based health authority. Almost all other health authorities in Canada have a regional rather then provincial focus. Through this survey we assessed the process of priority setting, the strengths and weaknesses to priority setting, improvements that could be made to make an explicit process feasible, organizational barriers that exist and facilitators that could be drawn upon to support an explicit approach.

The survey builds on previous studies in other jurisdictions which have examined decision maker views on aspects of priority setting. This type of approach has been shown elsewhere to be an important precursor to the development and implementation of an explicit approach to priority setting in health organizations [5,6,17]. Our results confirm the importance of gaining an understanding of organizational context prior to embarking on a new approach to priority setting, and add to the health policy literature by identifying key organizational barriers and facilitators to such activity.

### Examination of key results

At the time of the survey, a historical approach was the main mechanism for allocating scarce resources. This is similar to priority setting processes described in other health organizations in Canada, the United Kingdom, and Australia [5,6,17]. It is clear that decision makers are dissatisfied with this mechanism for allocating resources and desire a more explicit, evidence-based approach. This is not surprising as decision makers are being pressured to be more transparent and accountable in their decision making [18]. The recent literature on ethics suggests a growing interest in the fairness of priority setting processes [7,19,20].

Decision makers in the PHSA indicated that they would like more involvement from key stakeholders in their priority setting processes. They suggest that the optimal form of public involvement is through consultation in broad terms on issues of values and overall health priorities. This gives credence to findings from both Australia [5] and the recent Romanow Commission report in Canada which suggest that decision makers support a more general role for the public [21]. Organizational context also plays a role in how the public is engaged in decision-making. Abelson notes that organizations have the capacity to exert a strong "enabling" influence on public participation, the outcomes of which are dependent on the existence of a participatory culture and the amount of time that the culture has been in place [22].

With respect to physicians, decision makers supported a stronger physician role as the bearers of clinical evidence and the medical interpreters for management. Decision makers also felt that physicians would be in the best position to inform decision makers on what is considered best practice within each specialty, and to assist in discerning priorities accordingly. The role of physicians in management processes more generally has been a topic of much concern for a number of years [23,24]. Our results suggest several ways in which physicians can be involved in the area of priority setting and resource allocation. One key role for physicians in resource allocation would be to serve as the bearers of clinical evidence. It would also be beneficial for physicians to interpret the scientific evidence and translate that knowledge alongside of policy makers in resource allocation decisions. It should be noted that about one quarter of participants in our study (n = 8/25) were themselves physicians.

**Figure 2:**
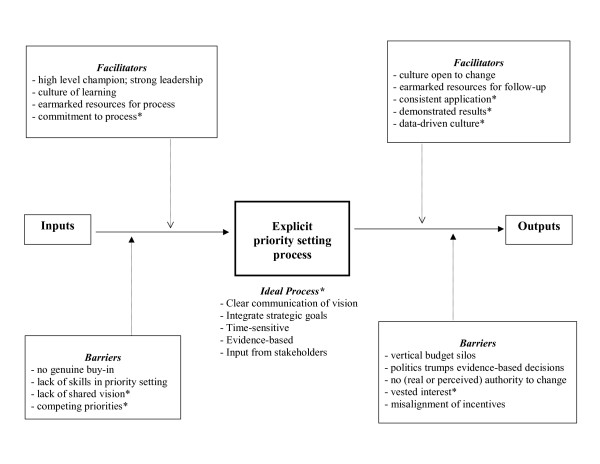
**PHSA organizational context model**. *Additions to model extending work from Mitton and Donaldson [19].

Finally, the decision makers in the PHSA had yet to undergo an explicit priority setting exercise, but were able to provide insight into the perceived barriers and facilitators to an explicit process (see Figure 2). The results can be divided into barriers and facilitators prior to embarking on an explicit priority setting process and those that impact the sustainability of recommendations. This model extends previous work on organizational behavior with respect to priority setting. Key factors for success include a lack of shared vision in priority setting, competing priorities, vested interest, the importance of demonstrated results, and a data-driven culture. While some of these parameters have been highlighted in other studies [8,17,25-27], we are adding to this literature the context of a provincial health authority and to our knowledge this is the first time that these additional factors have been presented in one empirical model.

### From theory to practice

Based on the findings from this survey, PHSA was not ready to engage in an explicit priority setting process that involves re-allocating resources across the organization. The primary rationale for this is the lack of integration that exists within the PHSA. According to Denis et. al. (2004), a sense of unified culture impacts the level of integration in an organization, which in turn impacts the ease by which priority setting can occur across the organization [28]. This can be attributed in part to infancy of the organization, the specialized nature of care in the PHSA, and strong history of each of the member agency. In addition, unlike regional health authorities which can often integrate services [29], it is more difficult between the agencies of the PHSA because of the specialized nature of care.

However, the desire of decision makers within the PHSA to adopt a formal approach to priority setting lead the Executive to develop a decision tool to impact prioritization of new service initiatives for the 2005/06 budget cycle. This tool, reported elsewhere [30], involved development and definition of eight key decision criteria and then rating a number of investment proposals against the criteria to derive an overall benefit score for each service option. Following its implementation in the first year, refinements were made and the tool was again employed for the 2006/07 budget cycle prioritization process. Key refinements included improvements to the criteria as well as greater process transparency through stronger communication efforts.

It is also important to note that in the current survey decision makers discussed that an explicit approach to priority setting could begin at the agency-level where resource re-allocation is likely to be more feasible and program managers are more familiar with shifting resources. These agencies could serve as 'change agents' [31], which over time can influence other parts of the organization. Some of this activity has been started, although formal follow-up is required to report activity in any detail. In moving forward, the key will be to continue to foster a transparent, consistent, and defensible approach. Ongoing education will also be required, and, in order for continued commitment, real results will have to be demonstrated.

### Limitations

The main limitation of this study is that we examined decision maker perceptions about priority setting before they had the opportunity to engage in an explicit process and reflect on its strengths and weaknesses. However, it was not the focus of this phase of the research to undertake an explicit priority setting case study. The purpose was to examine organizational context and other antecedent conditions related to priority setting, which were then utilized in later stages of the research as discussed in the preceding section.

### Transferability

This study was conducted in a provincial health authority in British Columbia. This health authority is the only one of its kind in Canada and the survey highlights some issues that are unique to the PHSA. However, this health authority faces many of the same issues found in other health organizations in Canada and elsewhere. For example, case studies in Australia, Alberta, and the UK describe difficulties in resource re-allocation and a desire for greater transparency in decision making. In comparing our results to findings elsewhere, it would seem that organizational context does not greatly differ between different types of health care organizations in different countries. Thus, the results of this study are likely transferable to other settings where decision makers have to make decisions amongst competing claims under constrained resources. This would include the above countries, and indeed Western Europe, and even the United States, where HMOs face similar constraints of resource scarcity and limited budgets [2].

## Conclusion

To date, there has been limited research pertaining to the organizational context within which difficult funding decisions are made. As well, few organizations have utilized this information to guide the development of explicit priority setting processes. The qualitative survey reported herein provides insight into the impact of context on an organization grappling with priority setting and was an important precursor to informing the next steps for developing an explicit approach in the PHSA. This work contributes to the growing body of literature on organizational behaviour and priority setting, and should be of value for decision makers and researchers interested in priority setting and resource allocation processes.

## Competing interests

JM is a paid employee of the Provincial Health Services Authority of BC.

## Authors' contributions

FT took the lead on drafting the paper. CM and JM made substantial intellectual contributions including input on study design, sample selection, questionnaire development and data analysis.

## Pre-publication history

The pre-publication history for this paper can be accessed here:


